# Advances in Nanoparticles as Non-Viral Vectors for Efficient Delivery of CRISPR/Cas9

**DOI:** 10.3390/pharmaceutics16091197

**Published:** 2024-09-11

**Authors:** Minse Kim, Youngwoo Hwang, Seongyu Lim, Hyeon-Ki Jang, Hyun-Ouk Kim

**Affiliations:** 1 Division of Chemical Engineering and Bioengineering, College of Art, Culture and Engineering, Kangwon National University, Chuncheon 24341, Republic of Korea; minse0415@kangwon.ac.kr (M.K.); dlatjsrb@kangwon.ac.kr (S.L.); 2 Department of Smart Health Science and Technology, Kangwon National University, Chuncheon 24341, Republic of Korea; hyw7066@kangwon.ac.kr; 3 Department of Systems Immunology, Division of Biomedical Convergence, College of Biomedical Science, Kangwon National University, Chuncheon 24341, Republic of Korea; 4 Multidimensional Genomics Research Center, Kangwon National University, Chuncheon 24341, Republic of Korea

**Keywords:** CRISPR/Cas9, delivery, nanoparticle, non-viral vector, viral vector

## Abstract

The clustered regularly interspaced short palindromic repeat (CRISPR)/Cas9 system is a gene-editing technology. Nanoparticle delivery systems have attracted attention because of the limitations of conventional viral vectors. In this review, we assess the efficiency of various nanoparticles, including lipid-based, polymer-based, inorganic, and extracellular vesicle-based systems, as non-viral vectors for CRISPR/Cas9 delivery. We discuss their advantages, limitations, and current challenges. By summarizing recent advancements and highlighting key strategies, this review aims to provide a comprehensive overview of the role of non-viral delivery systems in advancing CRISPR/Cas9 technology for clinical applications and gene therapy.

## 1. Introduction

Clustered regularly interspaced short palindromic repeats (CRISPRs) are repetitive nucleotide sequences arranged at regular intervals that function as adaptive immune systems in prokaryotes and provide protection against exogenous genetic elements. This immune defense is initiated through the acquisition and integration of 2–30 base pair (bp) segments of protospacers (which are derived from extrachromosomal elements such as bacteriophages) into the CRISPR loci. These integrated DNA fragments are termed “spacers”. Upon subsequent infection with bacteriophages of the same lineage, the stored spacers are transcribed into RNA. This RNA, in conjunction with CRISPR-associated proteins (Cas), guides the cleavage of viral DNA into sequences complementary to the spacer. The mechanism effectively neutralizes the invading viruses [1]. Among these systems, CRISPR/Cas9 has heralded a transformative era in genome editing. It provides significantly high accuracy and efficiency for target gene modification.

**Figure 1:**
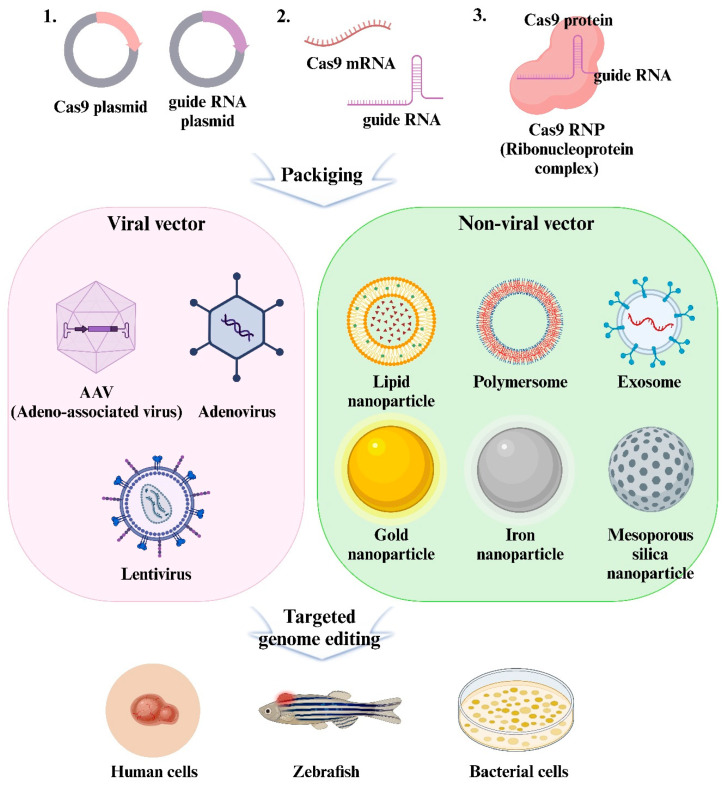
Scheme of delivery system of CRISPR/Cas9 using viral vectors and non-viral vectors encapsulated in nanoparticles.

However, the clinical application of CRISPR/Cas9 encounters significant challenges related to gene transfer. For CRISPR/Cas9 to achieve its therapeutic potential, it is crucial to deliver it efficiently and precisely to target cells or tissues while minimizing the off-target effects. This remains a key obstacle. The delivery vectors for CRISPR/Cas9 are generally categorized into viral and non-viral systems. Although viral vectors are effective, these pose limitations such as potential immunogenicity and the risk of introducing undesired mutations into the host genome. To address these issues, there is growing interest in developing non-viral delivery vectors through advancements in nanotechnology (Figure 1).

Nanoparticles are defined as particles with dimensions in the order of 10^−9^ m. These provide several advantages owing to their small size. Their chemical and physical properties are altered significantly because these have larger surface areas relative to their masses [2]. These size-dependent variations enable nanoparticles to exhibit distinctive behaviors including enhanced colloidal stability, ultrafast optical reactions, and novel electrical properties [3]. Because of these physicochemical properties, nanoparticles are rapidly emerging as high-potential modalities for the delivery of CRISPR/Cas9 [4]. Transporters with various nanoscale dimensions provide advantages such as customizable size and surface properties, biocompatibility, and the capacity to encapsulate and protect nucleic acid payloads [5]. Additionally, nanoparticles can enhance the cellular uptake of CRISPR/Cas9. Thereby, these overcome the obstacles associated with nucleic acid transfer and improve the efficiency of genome editing [6].

In this review, we aim to address the limitations associated with viral vectors in CRISPR/Cas9 delivery and evaluate recent advancements in nanoparticle-based delivery systems for non-viral vectors. We discuss the design principles, delivery mechanisms, applications, and clinical potential of various nanoparticle types including lipid-, polymer-, inorganic-, and extracellular vesicle-based nanoparticles. Our goal is to contribute to the development of safe, efficient, and clinically viable CRISPR/Cas9 delivery platforms by elucidating key strategies, challenges, and future directions in this rapidly evolving field.

## 2. Mechanism of CRISPR/Cas9 Genome Editing System

**Figure 2:**
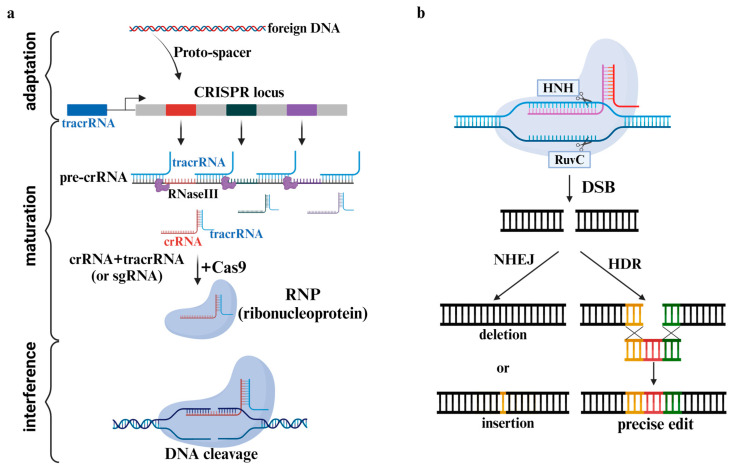
Mechanism of CRISPR. (**a**) Mechanism of CRISPR/Cas9 in cell. (**b**) DNA DSB repair system.

CRISPR/Cas9 is derived from the immune system of bacteria, identifies and cleaves specific DNA sequences, and contributes significantly to the development of gene-editing technology. This system operates in three steps: (1) spacer acquisition (adaptation), (2) maturation, and (3) interference (Figure 2a). Spacer acquisition begins with the identification of a protospacer adjacent motif (PAM) sequence in exogenous DNA derived from bacteriophages. In *E. coli* K12, Cas1 and Cas2 are the only proteins necessary for spacer insertion into the CRISPR locus. In addition, the coding or non-coding regions of DNA can be inserted. However, PAM sequences are essential [7,8]. Protospacer is a part of the DNA near PAM. It becomes prepared for maturation by being inserted into the CRISPR loci of the host genome. During the maturation step, pre-crRNA (pre-CRISPR RNA) is transcribed from the CRISPR repeat sequence and the inserted DNA [1]. Thereafter, transactivating crRNA (tracrRNA) and RNase III work in conjunction to form a mature crRNA by cleaving the pre-crRNA into spacer units [9]. These crRNA’s 5′ end contains the spacer that is complementary to protospacer, and the 3′ end contains a part of the CRISPR repeat sequences. A part of the CRISPR repeat sequence that exists at the 3′ end forms a crRNA–tracrRNA complex through base pairing with tracrRNA [10]. Mature crRNA–tracrRNA plays the role of guiding to the target gene as gRNA (guideRNA). gRNA exists as a ribonucleoprotein (RNP) that forms through binding with Cas9 [11]. Then, the interference step begins as the RNP searches for the PAM sequence adjacent to the target sequence in the genome. If the target sequence has PAM, as the double helix structure of the target DNA is unwound by the RNP, the gRNA binds to a strand of the target DNA to form a stable DNA–RNA hybrid structure that adopts the form of an R-loop [12]. The DNA–RNA hybrid structure thus formed is identified by Cas9 to cleave the target DNA.

Cas9 is a protein with a DNA cleavage function that consists of two lobes: nuclease (NUC) and recognition (REC) [13]. The NUC lobe consists of the HNH domain and the RuvC domain, which are directly involved in DNA cleavage. The REC lobe consists of RecI, II, and III, which are involved in target recognition [14]. These have alpha-helical structures and are similar to other known proteins [10]. If crRNA binds to the target DNA, the HNH domain cuts the complementary strand with the crRNA, and the RuvC domain cuts the opposite strand to form a blunt end [15]. Using Cas9 mutagenesis, mutations that eliminate the activity of the HNH domain (H840A) and Ruvc domain (D10A) were identified. This mutation converts Cas9 to nickase, which yields dead-Cas9 (dCas9), wherein the two domains are deactivated. Although dCas9 loses the cleavage activity of Cas9, the RNA-dependent DNA-binding capability remains [10]. Based on this, technology that controls the expression of specific genes has been developed by combining a transcription activator or repressor with dCas9 [16,17]. The overexpression system is named CRISPRa, and the underexpression system is named CRISPRi. Other studies demonstrated that CRISPRa and CRISPRi can regulate endogenous gene expression in mammalian cells [17,18,19]. Double-strand breaks (DSBs) in DNA caused by Cas9 can be repaired by the cell’s DNA repair pathway. This process occurs through nonhomologous end-joining (NHEJ) and homology-directed repair (HDR) [20] (Figure 2b). NHEJ is a repair mechanism that enables both ends of the cut DNA to stick together through the random insertion of a deletion (indel), which is mainly used to knock out genes. When DNA repair occurs through NHEJ, frameshift mutations or premature stop codons are formed, and the gene can be knocked out. Additionally, a few DSB can cause large deletions in the genome [21]. HDR is a DNA repair mechanism that occurs relatively less frequently than NHEJ because it can occur only in S or G2 phases and only if there is a donor template [22]. Donor templates generally use chromosomes that have homologous arms with a sequence complementary to the truncated portion. As sequences between homologous arms are inserted between DNA, gene knock-in or knock-out and accurate editing can be induced.

## 3. Advanced Gene Editing Tools Based on CRISPR/Cas9

In general, gene editing using Cas9 is intended for converting (point mutations), deleting, and inserting DNA base pairs (BPs). Although Cas9 is sufficient to induce stochastic indels, it has a limit in terms of inducing precise DNA conversion or insertion. Therefore, tools such as base editors and prime editors emerged with the modification of existing Cas9 or the addition of components [23]. Base editing is a gene editing technology that cuts only one strand of DNA. This is unlike Cas9, which causes double-strand breaks.

**Figure 3:**
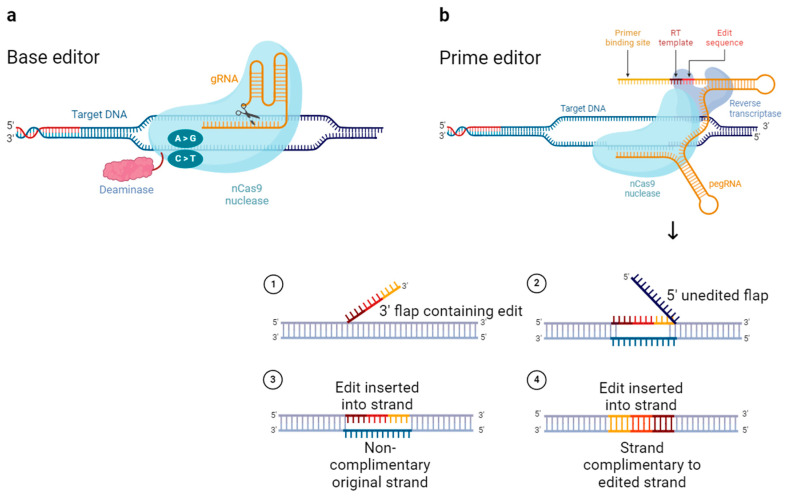
Base editor and prime editor Cas9 variants. (**a**) Scheme of base editor. Blue refers to nCas9. Deaminase of ABE is adenine deaminase, CBE is cytosine deaminase. (**b**) Scheme of prime editor. nCas9 fused with reverse transcriptase.

Because deaminase is attached to nCas9 (nickase Cas9), which has one side of its nuclease domain disrupted to cut only one DNA strand, it can convert only a single base (Figure 3a). The nick on the target strand is followed by deaminase deamination of a single base on the non-target strand. This is suitable for inducing PAM distal transition point mutations. Base editors are broadly divided into cytosine base editors (CBEs) and adenine base editors (ABEs). These convert cytosine to thymine through deamination, and adenine to guanine, respectively [24]. Because approximately half of pathogenic mutations in genetically related diseases are caused by single-base variations, base editors have a wide range of applications.

Prime editing is a gene-editing technology that does not cause double-stranded breaks and drives gene editing without a donor DNA template (Figure 3b). Prime editors use nCas9 with a disrupted nuclease domain (similar to base editors), but with the addition of reverse transcriptase (RT) rather than deaminase. When nCas9 causes single strand breaks in the non-target strand, the released 3′ end hybridizes with the 3′ end of a prime editing guide RNA (pegRNA) and is reverse transcribed by RT [25]. Prime editors are suitable for generating proximal point mutations, small insertions, and small deletions [24].

These modifications to Cas9 have been used to expand its range of applications and improve the gene-editing efficiency. However, because both base and prime editors are components added to the existing CRISPR/Cas9 system, they are relatively large. This limits their delivery.

## 4. Miniature CRISPR/Cas9

The commonly used *Streptococcus pyogenes*-derived Cas9 (SpCas9) is 1368 aa in size (Table 1). Smaller CRISPR/Cas9s are more favorable for transduction, and this has resulted in the identification of other miniature Cas9s in addition to SpCas9. *Staphylococcus aureus* Cas9 (SaCas9) is 1053 aa in size and identifies the 5′-NNGRRT PAM (R is A/G) [26,27,28]. *Neisseria meningitides* Cas9 (NmCas9) is 1082 aa in size and identifies the 5′-NNNNGATT PAM. These may be smaller in size because the length and structure of the wedge (WED), recognition (REC), and PAM interaction (PI) domains among the domains that comprise Cas9 differ from SpCas9 [29,30]. Although this size advantage is present, it limits the genes that can be targeted because relatively few PAM sequences can be identified. *Campylobacter jejuni* Cas9 (CjCas9) has a small size of 984 amino acids because of the significantly shorter length of its wedge (WED), recognition (REC), and PAM interaction (PI) domains compared to other Cas9s. CjCas9 identifies the 5′-NNNVRYM (V is A/G/C, R is A/G, Y is T/C, and M is A/C) PAM [31,32]. It is effective in eukaryotic cells. However, its editing efficiency is not remarkable. Additionally, similar to SaCas9 and NmCas9, it has a limited editing window owing to the relatively small number of identified PAM sequences [31]. Although the miniature Cas9 is small, the addition of fluorescent reporter genes or gRNAs required for delivery to the target can hinder packaging in a vector.

## 5. Clinical Trials of CRISPR System

CRISPR/Cas9-based clinical trials have been progressing for various genetic diseases, including β-thalassemia and sickle cell disease (SCD) (Table 2). Casgevy, an ex vivo therapy using CRISPR/Cas9 to treat transfusion-dependent β-thalassemia patients, received FDA approval on 8 December 2023. Inherited hemoglobin disorders are among the most common single-gene disorders worldwide. β-thalassemia is an autosomal recessive disorder caused by point mutations, indels, etc., in the human β-globin gene (*HBB*). It is characterized by a reduction in, or absence of, the β-hemoglobin chain [34,35]. The adult hemoglobin, hemoglobin A (HbA, α2β2), is composed of two α-hemoglobin chains and two β-hemoglobin chains. Here, the reduction in the β-hemoglobin chain causes an imbalance in the α/β globin ratio and the precipitation of free α-hemoglobin. This, in turn, can damage the cell membrane of red blood cells (RBCs) and cause anemia [34,35,36]. The strategies to treat this include mutation correction of the *HBB* gene through HDR, inhibition of α-globin gene (*HBA*) expression to balance the α/β globin ratio, and the induction of fetal hemoglobin (HbF, α2γ2) expression in hematopoietic stem and progenitor cells (HSPCs) or hematopoietic stem cells (HSCs) derived from β-thalassemia patients [37]. Among these, human HbF is the major hemoglobin in fetal RBCs and is almost completely replaced by HbA within a year by switching from γ-globin gene (*HBG*) to *HBB* gene expression [34,36]. Methods to increase the *HBG* expression include interfering with the HBG transcriptional repressor (B-cell lymphoma/leukemia 11A (BCL11A)) by targeting erythroid enhancers at the +58 site, which bind to the transcription factor GATA1 located +58 kb from the transcription initiation site of the *BCL11A* gene and increase BCL11A expression. Alternatively, interference with the BCL11A binding site in the *HBG1/2* promoter (115 bp upstream of the transcriptional initiation site) can replicate the hereditary persistence of fetal hemoglobin (HPFH) [38,39].

Sickle cell disease (SCD) is an autosomal recessive disorder caused by a single point mutation (*HBB*: c.20A > T) in the sixth codon of the *HBB* gene. It results in the substitution of hydrophilic glutamic acid for hydrophobic valine to form sickle-shaped erythrocyte anemia (HbS, βS). HbS is vulnerable to polymerization in the absence of oxygen, thereby forming long-chain polymers that distort the shape of RBCs. This makes these cells fragile, stiff, and incapable of deforming as these pass through narrow capillaries. This, in turn, causes vascular occlusion and hemolysis [34,40,41]. The treatment strategies include correction of *HBB* mutations through HDR and disruption of the *BCL11A* or *HBG1/2* promoters to reactivate HBG expression through NHEJ [42]. A novel approach to correcting HbS sickle-shaped mutations (*HBB*: c.20A > T) by ABE (T to C conversion) is under development. The development of a prime editor enables the correction of SCD-causing mutations in wild-type *HBB*, which can potentially cure SCD [43].

CRISPR-based gene editing technology can be applied not only to single gene disorders with clearly identified genetic predispositions, but also to polygenic disorders or diseases with unclear genetic causes, such as diabetes and hyperlipidemia. Type 1 diabetes is an insulin-dependent diabetes caused by autoimmune destruction of pancreatic islet β cells. Human pluripotent stem cell (hPSC)-derived β cell replacement therapy is effective. However, it is hindered by the immune rejection of transplanted cells and potential relapse of autoimmunity that impedes long-term success. CRISPR/Cas9-based multiplex genome editing can enable hPSCs or human embryonic stem cells to evade host adaptive and innate immune rejection upon cell transplantation. The editing targets include HLA, PD-L1, and chemokine ligand 10 (CXCL10) [44,45]. For example, hypoimmunogenic cell grafts for xenotransplantation can be generated by disrupting the *B2M* and class II transcriptional activating factor (*CIITA*) genes (key components of HLA classes I and II, respectively, in induced pluripotent stem cells (iPSCs)) and overexpressing CD47 to prevent phagocytosis by the host’s innate immune system [46]. Currently, two clinical trials (NCT05210530 and NCT05565248) sponsored by CRISPR Therapeutics and ViaCyte are being launched to evaluate the safety and tolerance of gene-edited cell replacement therapy in type I diabetic subjects [34].

## 6. Viral Delivery of CRISPR Reagents

### 6.1. Delivery Format of CRISPR/Cas9

The conventional approach for Cas9 delivery involves the delivery of plasmid DNA encoding Cas9 and gRNA. After the plasmid enters the nucleus, the components required for gene editing are transcribed and translated via cellular mechanisms [47]. This method can promote a stable and long-term expression of Cas9. This can be advantageous for gene editing. However, a risk of random insertion of plasmid DNA into the host genome exists. This unintentional insertion can cause off-target effects that influence the gene editing specificity. Plasmid DNA can also induce immunogenic responses by activating the cyclic GMP-AMP synthase (cGAS)-STING pathway, which identifies cytoplasmic DNA during transfection and induces inflammation and other immune responses [48]. The next step is to deliver Cas9 mRNA and gRNA. mRNA delivery is simpler because it bypasses the need for nuclear entry and transcription and facilitates direct translation into the cytoplasm [49]. Additionally, mRNA is susceptible to RNase degradation, which reduces the risk of mutations. However, the disadvantage is that mRNA instability can yield a lower transfection efficiency. The final method involves delivery in the form of RNPs. These are complexes of the Cas9 proteins and gRNA. The delivery of RNPs enables rapid entry into the cell and does not require nuclear entry of the component. This facilitates an immediate interaction with the target DNA in the nucleus. This, in turn, significantly reduces off-target effects owing to the transient expression and rapid degradation of components. RNPs can achieve a high editing efficiency because these are activated immediately after the delivery. However, the direct introduction of bacteria-derived Cas9 proteins into the body can activate an immune response and cause cytotoxicity. This poses challenges for their clinical application [50]. The delivery of Cas9 to the desired site at an appropriate concentration is a prerequisite for gene editing. Therefore, it is important to optimize the delivery format for the experimental conditions and to select the vector to introduce the delivery product.

### 6.2. Viruses Used for Viral Delivery 

#### 6.2.1. Adenovirus (AdV)

**Figure 4:**
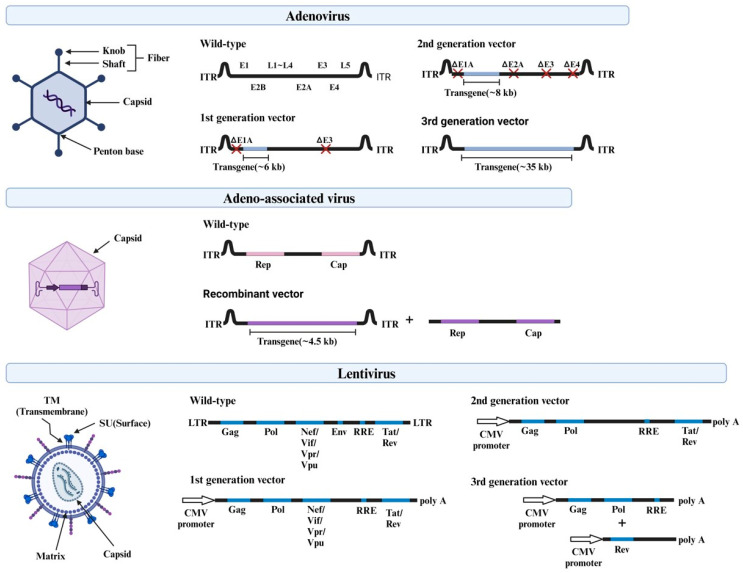
Structure of viral vectors. Structure and components of AdV, AAV, and LV. They are divided into different generations based on their components.

AdV, a non-enveloped virus with a double-stranded DNA genome, can be used as a vector to deliver CRISPR/Cas9 thanks to its inverted terminal repeat (ITR), which promotes DNA replication independent of the dodecahedral nucleocapsid (Figure 4). The *E1A* gene adjacent to the ITR, which is essential to initiate viral replication, and the *E3* gene, which is required for viral propagation, are key functions [51]. Three generations of AdV vectors have been used for gene therapy. The first generation accommodates a 6.5 kb transgene cassette with a deletion of the *E1/E3* region. The second generation additionally deletes *E2a*, *E2b*, or *E4* to provide a higher capacity and faster expression. The third generation retains only the ITR and the portion where the desired gene is to be introduced. This maximizes the capacity to 36 kb and thereby allows for the inclusion of multiple genes, including Cas9 and gRNAs (Table 3). AdV is present as an episome (a self-replicating genetic factor contained in chromosomes or in the cytoplasm) after infection. However, it is expressed transiently because it enters a latent state that does not produce functional viral particles. This reduces its immunogenicity. However, AdV particles can induce an innate immune response. This poses challenges such as potential tissue inflammation and complex production processes [52].

#### 6.2.2. Adeno-Associated Virus (AAV)

AAVs are single-stranded DNA viruses of the genus Dependoparvovirus in the family Parvoviridae. These are characterized by a small genome containing four open reading frames (ORFs) structured around a repetitive region (*rep*), a *cap*, and an ITR. ITR is a T-shaped structure at the end of the genome. It plays an important role in facilitating replication [51,52]. The rep genes encode non-structural proteins responsible for genome replication, transcriptional control, integration, and encapsulation. The cap genes encode the VP1, VP2, and VP3 proteins, which are essential for the structure formation of the virus. The infectious life cycle of AAV is divided into lytic and tropism phases. These are influenced by genome replication and the presence of helper viruses that determine whether viral genes are expressed. Acknowledged for their capability to infect both dividing and non-dividing cells, AAVs are favored for gene therapy owing to their low immunogenicity, potential for stable gene expression, and precise targeting capabilities [53] (Table 3). However, their limited replication capacity of approximately 4.7 kb hinders the introduction of Cas9, gRNAs, and promoters. This is generally resolved by splitting these into multiple vectors that are less efficient [51].

**Table 3:** Characteristics of viral vectors.

Type of Delivery System	Delivery Efficiency	Packaging Capacity	Major Advantages	Major Limitations	Reference
AdV	Medium	8~36 kb	Large packaging capacity; No-integration	Innate immune response	[52]
AAV	Medium	~4.7 kb	Low Immunogenicity; No-integration	Low packaging capacity	[54]
LV	High	~8 kb	Large packaging capacity; Efficient delivery	Random integration; High off-target effect	[51]

#### 6.2.3. Lentivirus (LV)

LVs are single-stranded RNA spherical viruses that have evolved over three generations and are used for gene delivery. The first generation retains a significant portion of the human immunodeficiency virus (HIV) genome, including essential genes, such as *gag* and *pol*; regulatory genes, such as *stat* and *rev*; and accessory genes, such as *vif*, *vpr*, *vpu*, and *nef*. The second-generation LV removes these accessory genes to improve the safety. The third generation further improves the safety and functionality by splitting critical genes into three plasmids: one containing *gag* and *pol*, another containing *rev*, and the third containing *env* [50]. With a replication capacity of approximately 8 kb, LVs can accommodate both Cas9 and gRNA within a vector (Table 3). This enables these to be transported across the nuclear pore. These are effective in both dividing and non-dividing cells. Additionally, these features render LVs particularly effective for infecting cells typically resistant to other methods [51]. However, there are ongoing concerns regarding the potential for viral genes to integrate randomly into the host genome. This can cause mutagenesis, off-target effects, and tumorigenesis, and thereby poses safety concerns.

### 6.3. Non-Viral Vectors to Overcome the Limitations of Viral Vectors

Viral vectors such as LV and AAV are commonly used to deliver CRISPR/Cas9 because of their strong binding and high in vivo efficiency. However, these vectors pose several challenges including the risk of undesired gene mutations, insertional mutagenesis, off-target effects, carcinogenicity, immunogenic reactions, and limited transfection volumes. In addition, the use of these vectors requires complex codon optimization and promoter selection for the effective insertion of the gene sequence. As an alternative, non-viral vectors are gaining traction. These are less immunotoxic and provide advantages in terms of efficiency, stability, specificity, and long-term gene expression.

Nanoparticle-based non-viral delivery systems are emerging as potential solutions to overcome the limitations associated with viral vectors (including the complex production processes, potential cell damage, and limited packaging capabilities) while reducing immunogenicity.

## 7. Non-Viral Vectors Delivery of CRISPR Reagents

Nanoparticle-based non-viral delivery systems present a valuable alternative to viral vectors for CRISPR reagent delivery. When designing nanoparticles for CRISPR/Cas9 delivery, several key factors must be considered. Stability is crucial to ensure that nanoparticles remain functional in physiological conditions until they reach target cells [4]. Targeting efficiency is important to enhance delivery specificity and minimize off-target effects [6]. The materials used should be biocompatible and non-toxic to avoid adverse immune responses, with biodegradable options preferred to prevent accumulation in the body [55,56,57]. Efficient encapsulation of CRISPR components is necessary for effective delivery, and strategies to facilitate endosomal escape are required to release the payload into the cytoplasm [58,59]. Additionally, the formulation should be scalable and reproducible to ensure consistent results, and safety considerations should address minimal cytotoxicity and immunogenicity. These considerations are essential for optimizing nanoparticle-based systems for CRISPR/Cas9 delivery, impacting the overall efficiency and safety of gene-editing applications.

When CRISPR reagents are delivered using nanoparticles designed with these factors in mind, they are encapsulated to enhance stability and protection against degradation. Designing nanoparticles to target specific tissues or cell types improves cellular uptake and reduces off-target effects. Nanoparticles can be composed of lipids, polymers, inorganic materials, or extracellular vesicles, each offering benefits like efficient gene transfer, biodegradability, and targeted delivery. Detailed information on these systems is shown in Table 4.

### 7.1. Non-Viral Delivery System

#### 7.1.1. Lipid-Based Nanoparticle

Lipid-based nanoparticles are acknowledged as effective delivery systems for CRISPR/Cas9 owing to their unique structure consisting of a hydrophilic head and hydrophobic tail. This structural composition enables these nanoparticles to efficiently combine with the cell membrane and facilitate the intracellular delivery of CRISPR/Cas9 to target cells through intracellular trafficking [76]. These nanoparticles facilitate the efficient encapsulation and transfer of both hydrophilic and hydrophobic molecules. Unlike conventional liposomes, cationic liposomes straightforwardly form complexes with nucleic acids through electrostatic interactions with negatively charged molecules such as CRISPR plasmid DNA, mRNA, and gRNA. These interactions protect nucleic acids from degradation and enhance their intracellular uptake during the critical stages of transcription and translation.

**Figure 5:**
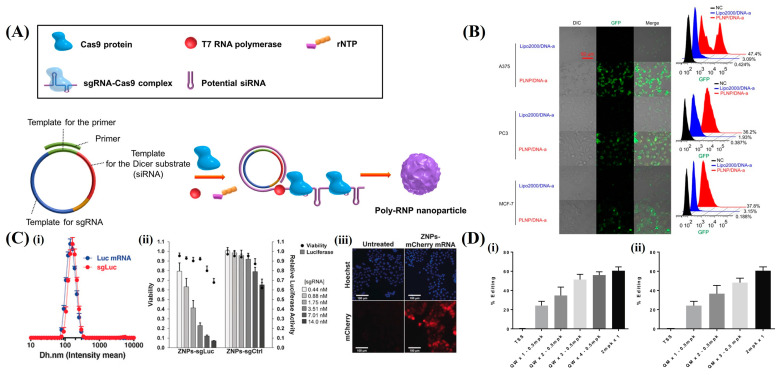
CRISPR/Cas9 delivery using lipid-based nanoparticles. (**A**) Polymeric sgRNA-Cas9 ribonucleoprotein (Poly-RNP) nanoparticles: schematic representation of the poly-RNP nanoparticle fabrication process using RCT. (**B**) In vitro transfection analysis of the CRISPR/Cas9 system via confocal laser scanning microscopy (CLSM) and flow cytometry (FCM). A375, PC3, and MCF-7 cells were transfected with Cas9-sgPLK–1a (DNA-a), Lipo2000/DNA-a, or PLNP/DNA-a, and images were captured 48 h post-transfection. (**C**) ZNPs for delivering long RNAs both in vitro and in vivo: (**i**) ZA3-Ep10 ZNPs (ZAL:cholesterol = 100:77:1 (mol); ZAL = 7.5:1 (wt)) are uniformly effective for both sgRNA and mRNA delivery. (**ii**) ZA3-Ep10 sgRNA ZNPs show dose-dependent Luc gene editing in HeLa-Luc-Cas9 cells. (**iii**) ZA3-Ep10 ZNPs can also deliver mCherry mRNA (observed after 18 h). (**D**) In vivo liver editing in CD–1 mice (n = 5/group) using LNPs co-formulated with Cas9 mRNA and a modified murine Ttr-specific guide: (**i**) Mice were dosed weekly with 0.5 mpk and sacrificed 1 week post-dosing; (**ii**) another group received monthly doses and was also sacrificed 1 week after the final dose. A positive control group received a single 2-mpk dose (black bar). Liver editing efficiency was assessed by NGS, with results shown as mean + SEM. Reproduced with permission from [55,60,63,82]; Copyright 2017, Elsevier; Copyright 2017, Springer Nature; Copyright 2016, Wiley Online Library; Copyright 2018, Elsevier.

Mout et al. developed a versatile method for the efficient cytosolic delivery of proteins by co-engineering proteins with an oligo(glutamate) sequence (E-tag) and arginine-functionalized gold nanoparticles (AuNPs). This method formed nanoassemblies that combined with cell membranes to release the proteins directly into the cytosol. This method successfully delivered five proteins that retained their activity for cancer cell killing, as demonstrated by Cre recombinase and granzyme A [77]. Zhang et al. developed a polyethylene glycol phospholipid-modified cationic lipid nanoparticle (PLNP) delivery system that encapsulated a CRISPR/Cas9/single-guide RNA (sgRNA) plasmid (Figure 5B). This system achieved up to 47.4% transfection efficiency in A375 cells and significantly downregulated polo-like kinase 1 (PLK–1) protein. As a result, it suppressed tumor growth by over 67% in melanoma-bearing mice. The PLNP-based approach demonstrated higher efficiency and safety than commercial transfection reagents [60]. By engineering a thermostable CRISPR/Cas9 system from Geobacillus stearothermophilus (iGeoCRISPR/Cas9) through directed evolution, Chen et al. achieved robust genome editing in cells and organs. These surpassed wild-type GeoCRISPR/Cas9 by over 100-fold in terms of performance. Intravenously injected iGeoCRISPR/Cas9 RNPs encapsulated in LNPs demonstrated significant editing efficiency in the liver and lung tissues of mice. This system provides a highly effective alternative to mRNA-based delivery. It enables efficient tissue-selective genome editing while minimizing toxicity [61].

These properties are utilized in commercial lipid-based transfection agents such as Lipofectamine™, Turbofect™, and Stemfect™. Additionally, these enhance the delivery and efficacy of DNA and RNA in vitro [78]. In particular, Lipofectamine™ has demonstrated the potential to evade lysosomal degradation and enhance transfection efficiency. This makes it a viable option for the delivery of CRISPR/Cas9 components [51]. 

One recent advancement in this area is Lipofectamine™ CRISPRMAX™. It is optimized for the delivery of CRISPR/Cas9/gRNA complexes and demonstrates superior gene editing efficiency with reduced cytotoxicity compared with conventional Lipofectamine™. Yu et al. investigated 60 transfection reagents across six mammalian cell lines. Lipofectamine CRISPRMAX emerged as the top performer, showing 40% and 15% higher genome modification efficiencies than Lipofectamine 3000 and RNAiMAX, respectively. The optimized conditions yielded maximum genome-editing efficiencies of 85%, 75%, and 55% in HEK293FT cells, mouse ES cells, and human iPSCs, respectively. Additionally, co-delivery with donor DNA resulted in up to 17% EmGFP-positive cells. Notwithstanding the robustness of Lipofectamine CRISPRMAX, neon electroporation was effective across various cell lines including difficult-to-transfect suspension cells. Owing to its convenience of use and low toxicity, researchers have recommended Lipofectamine CRISPRMAX for high-throughput drug screening and genome editing, where electroporation is less feasible [79]. Notwithstanding their in vitro efficiency, these lipid-based reagents encounter challenges in systemic delivery owing to safety concerns and limitations in their in vivo applications, primarily because of the size of the complexes formed [80].

To address the limitations of existing delivery methods, alternative strategies such as the electroporation of T lymphocytes and the fabrication of polymer gRNA complexes using rolling circle transcription (RCT) have been developed. RCT enhances stability and cellular uptake by generating long repeating RNA units that straightforwardly form complexes with cationic carriers [81]. 

Ha et al. developed a polymeric CRISPR/Cas9 system utilizing poly-RNP nanoparticles composed of a CRISPR/Cas9 endonuclease and polymeric sgRNA produced by RCT in conjunction with siRNA to enhance delivery efficiency. Figure 5A shows that, when delivered via cationic lipids, these nanoparticles generated multiple sgRNA-CRISPR/Cas9 RNP complexes through Dicer-mediated digestion of siRNA. This resulted in a more effective target gene disruption than monomeric complexes in cells and animal models. The poly-RNPs formed with chimeric poly-sgRNA/siRNA sequences further increased the gene disruption efficiency owing to the enhanced production of active RNPs post-delivery. This system demonstrated superior gene regulation in vivo using xenograft models. This, in turn, indicates its potential for various biomedical applications requiring precise gene disruption [82]. As shown in Figure 5C, Miller et al. developed zwitterionic amino lipids (ZALs) capable of non-viral co-delivery of long RNAs including CRISPR/Cas9 mRNA and sgRNAs, through ZAL nanoparticles (ZNPs). This delivery system achieved an over 90% reduction in protein expression in cells at low sgRNA doses and enabled permanent DNA editing with a sustained 95% decrease in protein expression. This was unlike transient RNAi therapies. ZNPs demonstrated a high efficiency in delivering mRNA both in vitro and in vivo, with successful intravenous co-delivery. This induced gene expression in multiple organs of engineered mice [63]. Finn et al. developed a biodegradable lipid nanoparticle (LNP) delivery system for CRISPR/Cas9. The results in Figure 5D indicate that significant in vivo genome editing with a single administration. This resulted in an over 97% reduction in transthyretin (Ttr) serum protein levels in mice, which was sustained for at least 12 months. This system uses chemically modified sgRNA for high activity. It is well tolerated and effective in both mouse and rat models. LNP systems showing a high level of durable gene editing are synthetic, extensible, and non-viral. Thus, these can potentially be utilized as treatment approaches for liver-based genetic diseases [55].

These polymer RNA nanoparticles demonstrated enhanced performances compared with monomeric RNA complexes. This is attributed to their increased efficiency in gene disruption when used in conjunction with the CRISPR/Cas9 protein [83]. Moreover, the incorporation of Dicer-sensitive siRNA sequences within these polymeric RNPs enhanced their in vivo stability and improved their target specificity [82]. 

Using the distinctive attributes of lipid-based carriers, delivery technologies are being optimized progressively to enhance efficiency and effectiveness. This effort is aimed at expanding the scope of gene therapy and ultimately maximizing the clinical potential of CRISPR/Cas9 technology.

#### 7.1.2. Polymer-Based Nanoparticles

**Figure 6:**
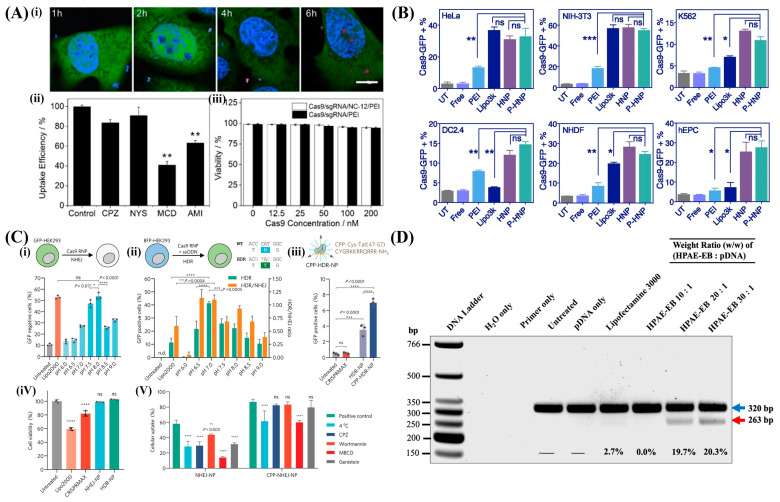
CRISPR/Cas9 delivery using polymer-based nanoparticles. (**A**) (**i**) Confocal microscopy images of U2OS.EGFP cells incubated with Cas9/sgRNA/NC–12/PEI for different time intervals, showing EGFP (green), Cas9 (red), and nuclei (blue). (**ii**) Analysis of Cas9/sgRNA/NC–12/PEI uptake in the presence of endocytosis inhibitors, with significant differences (** *p* < 0.01) compared to the control group. (**iii**) Flow cytometry assessment of cell viability in U2OS.EGFP cells after treatment with Cas9/sgRNA/NC–12/PEI or Cas9/sgRNA/PEI. (**B**) Transfection efficiency of Cas9-GFP plasmid px458 across various cell types using HNPs and P-HNPs, with comparisons to free plasmid and untreated groups. (**C**) In vitro studies on NHEJ-NPs and HDR-NPs: (**i**) optimization of pH for NHEJ-NP preparation in GFP-expressing HEK293 cells, with gene editing efficiency assessed by GFP loss via flow cytometry. (**ii**) pH optimization for HDR-NP preparation in BFP-expressing HEK293 cells, evaluating gene correction via GFP gain and gene disruption via BFP loss. (**iii**) HDR efficiency of CPP-HDR-NPs in BFP-expressing H9 hESCs, measured by GFP fluorescence. (**iv**) Cell viability of NHEJ-NP and HDR-NP in HEK293 cells via CCK–8 assay. (**v**) Endocytosis study of NHEJ-NP and CPP-NHEJ-NP in HEK293 cells, analyzing Cas9 RNP uptake with flow cytometry. (**D**) Excision efficiency of CRISPR-C7 plasmid in HEK293 cells, with densitometry analysis indicating up to 20.3% editing using HPAE-EB at a 30:1 ratio, compared to a commercial control. Significance level: * *p* < 0.05, ** *p* < 0.01, *** *p* < 0.001, and **** *p* < 0.0001. ns, not significant. Reproduced with permission from [59,65,88,92]; Copyright 2015, Wiley Online Library; Copyright 2018, National Academy of Science (PNAS); Copyright 2022, Wiley Online Library; Copyright 2022, Springer Nature.

Polymer-based nanoparticles are non-viral delivery systems for CRISPR components that provide advantages such as a low immunogenicity, high biocompatibility, and biodegradability. These nanoparticles can utilize a broad array of chemical diversity and functionalization potential to create various structures. This enables precise control over their properties [56]. Owing to this flexibility, polymer-based nanoparticles are well-suited for a range of applications, including encapsulating drugs within their core, chemically conjugating drugs to the polymer matrix, and attaching drugs to their surfaces. This flexibility is particularly advantageous for gene editing transfer techniques because it enables the efficient delivery of both hydrophilic and hydrophobic macromolecules, including proteins. Among these, polymers such as polyethyleneimine (PEI), poly(L-lysine) [84], chitosan [85], and poly(amidoamine) (PAMAM) [86] have attracted significant attention in recent studies because of their efficacy in delivering gene-editing tools. Kang et al. covalently modified CRISPR/Cas9 with cationic polymers to form nanosized CRISPR complexes (Cr-nanocomplexes), maintained CRISPR/Cas9’s functionality, and achieved efficient double-stranded DNA cleavage. Targeting the mecA gene in methicillin-resistant Staphylococcus aureus (MRSA), the Cr-nanocomplex demonstrated significantly higher genome editing efficiency than native CRISPR/Cas9 or lipid-based formulations [87]. Liu et al. developed a targeted therapy using a CRISPR/Cas9 system encapsulated in PEG-PLGA-based cationic lipid-assisted polymeric nanoparticles (CLANs). These nanoparticles carry a CRISPR/Cas9 plasmid targeting the BCR-ABL fusion gene. These specifically disrupted the fusion gene without affecting the normal BCR and ABL genes. The intravenous administration of these CLANs in a CML mouse model effectively knocked out BCR-ABL and improved survival. This demonstrates a potential strategy for targeted CML treatment using CRISPR/Cas9 and nanocarriers [64]. Sun et al. developed DNA nanoclews (NCs). These are yarn-like DNA nanoparticles synthesized by rolling circle amplification to efficiently deliver CRISPR/Cas9 protein and sgRNA complexes into human cell nuclei for targeted gene disruption while maintaining cell viability. As depicted in Figure 6A, optimal editing was achieved when the DNA nanoclew sequence partially complemented the sgRNA guide sequence, thereby enhancing the delivery efficiency. This versatile system (the polymeric nanoparticle for CRISPR/Cas9 delivery) stabilizes the CRISPR/Cas9/sgRNA complex and may be adapted for multiplex editing, cell-specific targeting, and homology-directed repair [65]. Wang et al. developed PEGylated nanoparticles (P-HNPs) based on the cationic α-helical polypeptide poly(γ–4-((2– (piperidin–1–yl) ethyl) aminomethyl) benzyl-l-glutamate) to enhance the delivery of CRISPR/Cas9 gene-editing components. These nanoparticles facilitated the cellular uptake, endosomal escape, and nuclear transportation of CRISPR/Cas9 plasmids and sgRNAs. Figure 6B reveals that P-HNPs achieved up to 60% CRISPR/Cas9 transfection efficiency and 67.4% sgRNA uptake. Therefore, these outperformed existing polycation-based systems. They enable effective single or multiplex gene editing in various cell types and tumor tissues, with maximum in vitro editing efficiencies of 47.3%. In vivo, P-HNPs targeting the Plk1 gene in HeLa tumor cells resulted in 35% gene deletion, a 66.7% reduction in Plk1 protein levels, tumor growth suppression of over 71%, and improvement of animal survival rates to 60% within 60 days. This versatile, nonviral gene-editing platform displays potential for biological research and therapeutic applications with its advantages in membrane penetration and gene-editing efficiency [59].

Xie et al. developed the pH-responsive polymer nanoparticles NHEJ-NP and HDR-NP. These are capable of efficiently delivering CRISPR/Cas9 RNPs and donor DNA for gene disruption and correction. NHEJ-NPs (29.4 nm) and HDR-NPs (33.3 nm) were generated through simple mixing, pipetting, and vortexing processes. These demonstrated high payload capacity and uniform sizes. These nanoparticles were tested in vivo and showed efficient gene editing in mouse liver, lung, and skeletal muscle through intravenous, intratracheal, and intramuscular injections, respectively (Figure 6C). Specifically, HDR-NPs restored muscle strength in a Duchenne muscular dystrophy mouse model by increasing dystrophin expression. The nanoparticles displayed good biocompatibility, low immunogenicity, and high stability when stored at various temperatures. Their convenience of synthesis, storage, and transportation, in conjunction with their high editing efficiency, emphasizes their potential for clinical translation [88]. 

Notwithstanding their numerous advantages, cationic polymer-based nanoparticles may present toxicity concerns. Their application is constrained by the limited number of formulations approved by regulatory bodies such as the FDA [89]. To address these issues, research is ongoing to modify these polymers or to combine them with other substances such as lipids to enhance their safety and efficacy [90]. For example, polyethylene glycol (PEG), an FDA-approved hydrophilic polymer, is used to coat cationic polymers. This modification extends the circulation time in the bloodstream and enhances stability, biocompatibility, and biodegradability [57].

The practical application of these polymer-based systems has been validated by several studies. These have successfully facilitated the transfer of the CRISPR/Cas9/gRNA RNP complex into the human nucleus [91]. These systems have demonstrated reduced toxicity and high editing efficiency compared with lipid-based systems. As shown in Figure 6D, O’Keeffe Ahern et al. demonstrated the potential of a highly branched poly(β-amino ester) polymer, HPAE-EB, for CRISPR/Cas9 delivery. It achieved 15–20% genomic excision in HEK293 cells and over 40% in human recessive dystrophic epidermolysis bullosa (RDEB) keratinocytes using CRISPR/Cas9 RNPs. The high encapsulation efficiency and favorable biophysical properties of HPAE-EB make it a potential alternative to other viral vectors. Although initial plasmid-based strategies showed limited success, RNP delivery significantly improved gene editing efficiency and collagen VII restoration [92]. Polyethyleneimine has demonstrated enhanced performance in CRISPR-mediated gene editing in vitro [93]. Meanwhile, polyethylene-based polymer carriers have enabled in vivo investigations of tumor suppressor genes [94]. Yue et al. developed graphene oxide (GO)-based nanocarriers combined with polyethylene glycol (PEG) and PEI for the direct delivery of high-molecular-weight CRISPR/Cas9/sgRNA complexes. These achieved a gene editing efficiency of approximately 39% in human AGS cells. This system protects the sgRNAs from enzymatic degradation and ensures high stability, which is essential for in vivo applications. The GO-mediated delivery system facilitated the endocytosis, endosomal escape, and nuclear entry of CRISPR/Cas9/sgRNA complexes. This presents a potential new approach for biomedical research and targeted gene engineering [58].

Additionally, amine-terminal polyamide-modified dendrimers and pegylated chitosan nanocomposites have been shown to be effective in CRISPR-mediated genome editing across various cell lines [95]. Tomalia et al. developed a new class of topological macromolecules called “starburst polymers”. These were synthesized by chemically bridging dendrimers, which are highly branched, symmetrical, and have reactive end groups. These dendrimers were created using time-sequenced propagation techniques, and allow for controlled molecular weight, branching, and versatility in terminal-group design. The unique architecture of dendrimers enables the potential construction of biocatalytic mimics and provides models for studying fractal and uniform network polymer topologies [96]. Liu et al. developed a dual-target delivery system for the CRISPR-CRISPR/Cas9 plasmid to achieve efficient genome editing in target tumor cells and facilitate field detection of related protein expression. This system uses polymer/inorganic hybrid nanoparticles encapsulating the CRISPR/Cas9 plasmid for CDK11 knockout. It features a core of protamine sulfate, calcium carbonate, and calcium phosphate. The nanoparticle surface is decorated with biotinylated carboxymethyl chitosan and AS1411 aptamer-incorporated carboxymethyl chitosan for targeted delivery to tumor cells and nuclei. The approach resulted in an over 90% reduction in CDK11 protein levels and significant downregulation of other tumor-related proteins such as MMP-9, VEGF, and survivin while upregulating p53. Additionally, it enabled in situ monitoring of mRNA levels and distribution within subcellular organelles [66]. This success emphasizes the potential of polymer-based nanoparticles as highly effective CRISPR/Cas9 delivery tools. These provide potential alternatives to viral vectors and expand the scope of gene therapy applications.

#### 7.1.3. Inorganic Nanoparticles

Inorganic nanoparticles in the field of nanotechnology have become remarkable candidates for both therapeutic and diagnostic purposes. This is because of their diverse applications and unique properties such as convenient synthesis methods and variations, tunable sizes, and reactivity to light irradiation [97]. These nanoparticles are composed of materials such as gold, silica, and iron. These have been engineered into various sizes, structures, and shapes to address specific requirements [98]. 

##### Gold Nanoparticles (AuNPs)

AuNPs can be utilized in various forms, including nanopores, nanorods, nanoshells, and nanocages [99]. The intrinsic physicochemical properties of AuNPs make these suitable for the delivery of CRISPR/Cas9 [100]. The photothermal properties of AuNPs originate from the vibrations of free electrons on their surfaces. These enable precise targeting and delivery [101]. Additionally, their small size and structural flexibility enhance their capability to efficiently penetrate the plasma membrane. This improves the delivery effectiveness while minimizing the lipid bilayer disruption [102]. Tarach et al. presented a multifunctional delivery system for CRISPR/Cas9 gene editing. It was designed to enhance the delivery efficiency for tumor therapy using these photothermal effects. The PEG-lipid/AuNPs/CRISPR/Cas9-sgPlk–1 (LACP) system combines TAT peptide-modified AuNPs to condense CRISPR/Cas9 plasmids with lipid encapsulation for stability and cellular uptake. The laser-induced thermal effects of AuNPs enable the release of CRISPR/Cas9 into the cytosol. TAT peptides guide the plasmids into the nucleus, thereby effectively targeting and knocking out Plk–1 in tumor cells. This method demonstrated significant tumor inhibition in vitro and in vivo. Thus, it is a potential tool for targeted gene editing and the treatment of various diseases [95]. Moreover, by leveraging the plasmonic properties of AuNPs, which enable tissue- and cell-type-specific targeting, we can enhance the delivery precision and control the transient activation. This would minimize the off-target effects [103]. 

Lee et al. developed CRISPR-Gold. It is a novel delivery vehicle composed of AuNPs conjugated to DNA and complexed with cationic endosomal-disruptive polymers. The system successfully delivered the CRISPR/Cas9 RNP and donor DNA into various cell types. Thereby, it achieved an efficient correction of Duchenne muscular dystrophy (DMD) mutations in mice through local injection, with minimal off-target effects. CRISPR-Gold provides a potential non-viral approach for HDR-based gene editing and thereby represents a potential strategy for treating genetic disorders such as DMD by correcting mutations and regenerating functional proteins [68].

**Figure 7:**
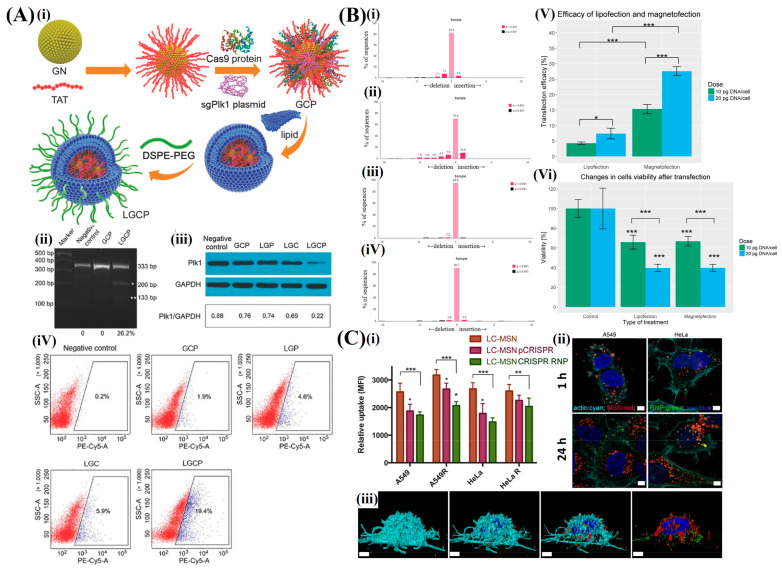
CRISPR/Cas9 delivery using inorganic nanoparticles. (**A**) Polymeric sgRNA-Cas9 ribonucleoprotein (Poly-RNP) nanoparticles: (**i**) schematic illustration of Poly-RNP nanoparticle fabrication using reversible addition-fragmentation chain transfer (RCT) polymerization. (**ii**) T7E1 assay results showing cleavage of the Plk1 locus in A375 cells induced by LGCP, with mutation frequency calculated from cleaved band intensity. (**iii**) Western blot analysis of Plk1 expression in A375 cells treated with LGCP, indicating the impact on protein levels. (**iv**) Flow cytometry (FCM) analysis of apoptosis in A375 cells treated with LGCP, with positive apoptotic cells identified within the frame. (**B**) Evaluation of CRISPR/Cas9-induced indels and cell viability: (**i**) and (**ii**) analysis of indel patterns in cells transfected via magnetofection at 10 and 20 pg DNA/cell, respectively. (**iii**) and (**iv**) Comparison with lipofection at the same DNA doses using TIDE software; The sequencing data were analyzed by Tracking of Indels by Decomposition (TIDE) web tool (https://tide.deskgen.com/), initially described by Brinkman and colleagues, showing higher editing efficiency for magnetofection. (**v**) CRISPR/Cas9 genome editing efficacy, with magnetofection significantly outperforming lipofection. (**vi**) Cell survival rates were similar between magnetofection and lipofection, with no significant differences in viability. (**C**) Uptake of lipid-coated mesoporous silica nanoparticles (LC-MSN): (**i**) quantification of LC-MSN uptake in A549 and HeLa cells using flow cytometry, showing significant differences in fluorescence intensity. (**ii**) Confocal microscopy images of cells treated with RNP-loaded LC-MSN for 1 or 24 h. (**iii**) 3D surface-rendered confocal images showing intracellular localization of nanoparticles in A549 cells after 1 h of incubation. Scale bars = 5 µm. Significance level: * *p* < 0.05, ** *p* < 0.01, *** *p* < 0.001. Reproduced with permission from [70,104,107]; Copyright 2017, Wiley Online Library; Copyright 2019, Springer; Copyright 2020, Elsevier.

Wang et al. reported a novel strategy to deliver CRISPR/Cas9 protein and a single-guide RNA (sgRNA) plasmid using a nanocarrier with a gold nanocluster (GN) core and lipid shell (Figure 7A). AuNPs (GNs) were modified with an HIV–1 transactivator of transcription peptide (TAT). This enabled the delivery of the CRISPR/Cas9/sgRNA complex into the cell nuclei. The unique properties of GNs, including biocompatibility, convenience of functionalization, and efficient cellular uptake, make these remarkable options for nonviral delivery systems. This approach was used to treat melanoma by designing sgRNA targeting Polo-like kinase–1 (Plk1) in a tumor. The nanoparticle formulation (polyethylene glycol lipid/GNs/CRISPR/Cas9 protein/sgPlk1 plasmid, LGCP) downregulated Plk1 protein expression in A375 cells in vitro. Furthermore, LGCP suppressed melanoma progression by 75% in mouse models [104].

Lee et al. demonstrated the capability to edit genes in the brain of adult mice by intracranial injection of CRISPR-Gold, a non-viral delivery system of CRISPR/Cas9 RNPs. This system efficiently delivered both CRISPR/Cas9 and Cpf1 RNPs to major brain cell types including neurons, astrocytes, and microglia, with minimal toxicity. Notably, CRISPR-Gold targeting of the metabotropic glutamate receptor 5 (mGluR5) gene significantly reduced mGluR5 levels in the striatum, and was shown to reverse repetitive behaviors in a mouse model of fragile X syndrome (a form of autism spectrum disorder) [67].

Notwithstanding the generally low toxicity of AuNPs, concerns remain regarding the potential toxicity of other nanomaterials and risk of substantial metal accumulation in vivo. Therefore, further studies are required to thoroughly assess their safety [105]. 

##### Magnetic Nanoparticles

Magnetic iron oxide nanoparticles display superparamagnetic properties. This renders these highly suitable for applications involving heat-based treatments [106]. These nanoparticles may be particularly advantageous for enhancing the in vivo activity of CRISPR/Cas9 when subjected to magnetic fields. This provides a novel approach for more precise control of the gene-editing process [69]. Hryhorowicz et al. evaluated the use of magnetic nanoparticles and a magnetic field gradient to enhance the delivery of CRISPR/Cas9 constructs to porcine fetal fibroblasts. Polyethylenimine-coated magnetic iron-oxide nanoparticles were used to form magnetic plasmid DNA lipoplexes. The CRISPR/ Cas9 construct was designed to induce site-specific cleavage of the porcine H11 locus. As detailed in Figure 7B, quantitative analysis of genomic cleavage using sequence trace decomposition demonstrated that the magnetofection efficiency was over 3.5 times higher than that for conventional lipofection methods. The tracking of indels using the decomposition web tool accurately determined the spectrum of the indels that occurred. Furthermore, no additional cytotoxicity associated with the use of the MNPs was observed. These results indicated that magnetofection effectively delivers CRISPR/Cas9 constructs to porcine fetal fibroblasts with minimal cell toxicity [107].

Zhu et al. developed a system using magnetic nanoparticles (MNPs) complexed with recombinant baculoviral vectors (BVs) for localized activation of CRISPR/Cas9. The magnetic field enhanced the cellular uptake of MNP-BVs. This allowed for targeted gene delivery and transient CRISPR/Cas9 expression, specifically in the desired tissue. This approach leverages the high cargo capacity of BVs and their capability to circumvent systemic inactivation. Thereby, it ensures that gene editing is constrained to the target area while preventing inactivation in other tissues [69]. Rohiwal et al. developed CRISPR/Cas9 complexed PEI MNPs and evaluated their efficiency in a stable HEK293 cell line expressing a traffic light reporter (TLR–3) system. This approach enables the assessment of homology-directed repair (HDR) and nonhomologous end-joining (NHEJ) events following transfection with nanoparticles. The MNPs synthesized via co-precipitation had an average particle diameter of approximately 20 nm. Dynamic light scattering and zeta potential measurements indicated that the nanoparticles exhibited a narrow size distribution and adequate colloidal stability. The genome editing efficiency of the MNPs was comparable to that achieved using standard lipofectamine transfection methods. This study demonstrated the feasibility of using non-viral delivery systems to deliver CRISPR/Cas9 components and DNA templates. This facilitates both HDR and NHEJ within the same assay. Our results indicate that PEI-MNPs represent a potential delivery system for plasmids encoding CRISPR/Cas9 and template DNA. This potentially improves the safety and efficacy of gene-editing applications [108].

##### Mesoporous Silica Nanoparticles (MSNs)

Additionally, MSNs display distinctive physicochemical properties owing to their porous structure, which facilitates the encapsulation and controlled release of therapeutic agents [70]. MSNs can accommodate a broad spectrum of molecular sizes ranging from small drugs to large biomolecules. Their low toxicity and high biocompatibility make these suitable for targeted and stimulus-responsive drug delivery, particularly in combination therapies for tumors [71]. Figure 7C demonstrates that Noureddine et al. presented a novel lipid-coated mesoporous silica nanoparticle (LC-MSN) designed for CRISPR gene delivery. It effectively loaded and released CRISPR components, thereby achieving up to 70% release within cancer cells. The LC-MSNs can transport up to 145 µg of RNP or 40 µg of plasmid per mg of nanoparticles. Moreover, these have demonstrated approximately 10% gene editing efficiency in both in vitro cancer cell lines and an in vivo reporter mouse model. These nanoparticles remain stable in ethanol for at least two years and dissolve up to 25% in human plasma within three days. This provides a potential non-viral delivery system with good cellular compatibility and efficient endosomal transportation. Notwithstanding the moderate editing efficiency, the system’s chemical adaptability and versatile delivery capabilities provide a strong foundation for further optimizing gene-editing applications [70].

Lipid-coated mesoporous silica nanoparticles (LCMSNs) demonstrate a high potential for delivering CRISPR/Cas9 RNPs to address viral infections and thereby address the challenges of flexible and rapid therapeutic development against high-pandemic-risk pathogens. LaBauve et al. reported the successful delivery of RNPs targeting the Niemann–Pick disease type C1 gene (NPC1), which is crucial for Ebola virus entry, using LCMSNs. This resulted in an efficient reduction in viral infection in vitro and in vivo, specifically in mouse liver tissue via systemic administration. The stellate nanoparticles with DCD3330 lipid-coating were demonstrated to be the most effective. This indicated their therapeutic potential in targeting host genes essential for pathogens, such as EBOV. Further optimization and evaluation of new CRISPR technologies such as CRISPRinhibition and CRISPRoff are necessary to enhance their safety and efficacy for broader applications [71]. 

Additionally, their scalability and surface functionalization capabilities continue to advance the research on their potential as nanocouriers. This has resulted in significant improvements in gene therapy and nanocoupling systems [109]. Ongoing research is crucial for developing groundbreaking treatments and diagnostic solutions as efforts to identify optimal nanoparticle carriers persist.

#### 7.1.4. Extracellular Vesicles-Based Nanoparticles

Exosomes are natural membrane-bound vesicles derived from the endoplasmic reticulum. These have been studied extensively as potential carriers of CRISPR/Cas9 components because of their intrinsic biocompatibility and low immunogenicity [110]. Engineered exosomes have been investigated extensively for their capacity to transport various biomolecules including plasmids, proteins, siRNAs, and miRNAs. This has positioned these as potential candidates for cell-free therapies [111]. Several recent studies have emphasized the potential for using exosomes as targeted delivery vehicles for gene editing tools.

A key advantage of exosome-based delivery is its capability to encapsulate functional gRNA and CRISPR/Cas9 proteins. This effectively facilitates the intracellular delivery of CRISPR/Cas9 gene-editing activity [112]. Exosomes have been engineered to deliver CRISPR/Cas9 components to target cells using bioengineered Vero and Chinese hamster ovary (CHO) cells. This demonstrates their potential as effective gene-editing mediators [113]. Furthermore, exosomes derived from tumor cells display a natural affinity for maternal cells. This facilitates the efficient targeting and delivery of CRISPR/Cas9/gRNA to tumor tissues. Research has demonstrated that exosomes from various cancer cell lines can accumulate in tumor tissues. This would effectively inhibit target gene expression and induce apoptosis in cancer cells [114]. This targeted delivery strategy provides noteworthy prospects for advancements in cancer gene editing and treatments. To overcome the challenge of incorporating larger DNA fragments into exosomes, hybrid exosomes have been engineered by combination with liposomes or other nanomaterials [115]. This approach significantly enhances the loading efficiency of plasmid DNA, thereby enabling the transfection of cells that are conventionally challenging. Hybrid exosomes provide a versatile platform for delivering CRISPR/Cas9 components. This has broadened the potential of exosome-based gene-editing technologies. In addition, a novel method that enables efficient loading and delivery by combining CRISPR/Cas9 mRNA and proteins with exosomes has been developed [116]. Exosomes have significantly enhanced their potential as nucleic acid carriers for gene editing by incorporating protein domains that bind to RNA or by effectively encapsulating CRISPR/Cas9 mRNA and gRNA using cellular nanoporation techniques. 

**Figure 8:**
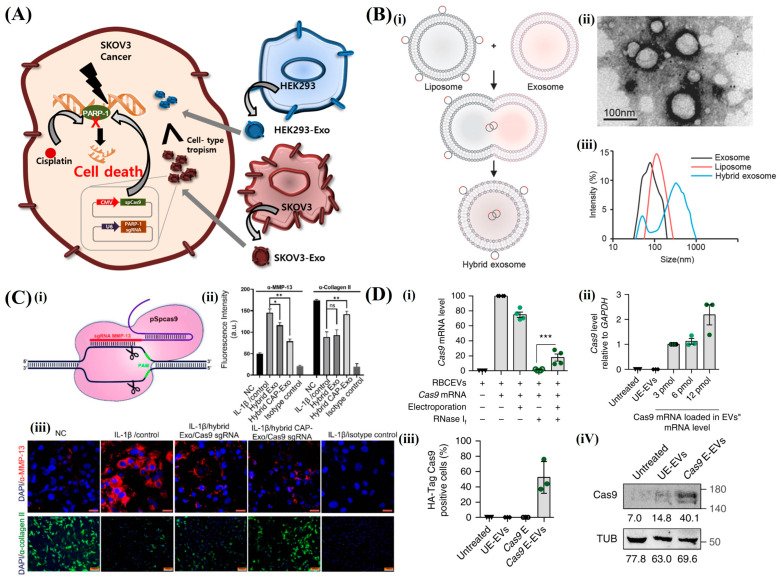
CRISPR/Cas9 delivery using extracellular vesicles-based nanoparticles. (**A**) Combination therapy in ovarian cancer cells. Schematic of loading Cas9 and PARP–1 sgRNA into tumor-derived exosomes (SKOV3-Exo) using electroporation. SKOV3-Exo shows enhanced targeting of SKOV3 tumor cells compared to HEK293-Exo. (**B**) Hybrid exosomes for plasmid selivery. (**i**) Schematic of hybrid exosome production by fusing isolated exosomes with liposomes. (**ii**) Electron microscopy image of hybrid exosomes after 12 h of fusion (scale bar: 100 nm). (**iii**) Size distribution analysis of exosomes, liposomes, and hybrid exosomes. (**C**) Exosome-mediated Cas9 delivery to chondrocytes. (**i**) Design of Cas9 sgMMP–13 system for targeting MMP–13 in chondrocytes. (**ii**) Quantification of the fluorescent signals. (**iii**) Immunofluorescence images of MMP–13 expression and collagen degradation in different treatment groups (scale bar: 20 µm for MMP–13; 100 µm for collagen II). (**D**) RBCEVs for Cas9 mRNA delivery to leukemia cells. (**i**) Cas9 mRNA levels in RBCEVs with and without electroporation, and RNase If treatment. (**ii**) Relative Cas9 mRNA levels in MOLM13 cells treated with electroporated RBCEVs. (**iii**) Percentage of MOLM13 cells positive for HA-Cas9 protein. (**iv**) Western blot of Cas9 and α-tubulin in MOLM13 cells, with quantification and expression changes of miR–125b and BAK1.Significance level: * *p* < 0.05, ** *p* < 0.01, *** *p* < 0.001. ns, not significant. Reproduced with permission from [73,75,117,118]; Copyright 2017, Elsevier; Copyright 2018, Wiley Online Library; Copyright 2022, National Institutes of Health (NIH); Copyright 2018, Springer Nature.

McAndrews et al. presented a novel non-viral delivery system for CRISPR/Cas9 based on engineered exosomes that addresses key issues such as immunogenicity, limited packaging capacity, and low tolerance. The approach utilizes non-autologous exosomes to encapsulate CRISPR/Cas9 plasmid DNA. This enables targeted gene editing in cancer cells. Our proof-of-principle studies demonstrated that exosomes loaded with CRISPR/Cas9 effectively target the KrasG12D oncogene in pancreatic cancer. This results in suppressed cell proliferation and reduced tumor growth in both subcutaneous and orthotopic models [74]. Cancer-derived exosomes have emerged as effective low-immunogenicity carriers for CRISPR/Cas9 delivery. This provides a potential solution for genome editing in cancer therapy. Unlike epithelial cell-derived exosomes, cancer-derived exosomes exhibit improved targeting and accumulation in ovarian cancer tumors. This is evidenced in SKOV3 xenograft models. Kim et al. used these exosomes to deliver a CRISPR/Cas9 plasmid to inhibit poly(ADP-ribose) polymerase–1 (PARP–1) (Figure 8A). This caused apoptosis and improved the chemical sensitivity to cisplatin. The approach disrupts PARP–1 and synergistically increases the efficacy of chemotherapy. This, in turn, emphasizes the potential of tumor-derived exosomes for advanced cancer treatment [75]. Lin et al. developed hybrid exosomes by incubating exosomes with liposomes. According to Figure 8B, this resulted in an efficient encapsulation of large plasmids including CRISPR/Cas9 expression vectors. These hybrid nanoparticles successfully delivered the CRISPR/Cas9 system to mesenchymal stem cells (MSCs), which could not be transfected with liposomes alone. This emphasized their potential for in vivo gene manipulation. This new delivery strategy addresses the challenges of efficient and safe CRISPR/Cas9 delivery. Thereby, it provides an alternative to viral vectors such as AAV, which involve immunogenicity and long-term expression concerns. This, in turn, enhances the prospects for gene therapy applications, including the treatment of genetic diseases such as Duchenne muscular dystrophy [117]. Liang et al. presented a cell-specific delivery system for gene editing using hybrid exosomes combined with liposomes to target chondrocytes in the articular cartilage. The engineered exosomes were enhanced with a chondrocyte affinity peptide (CAP). These effectively encapsulated CRISPR/Cas9 plasmids. When administered intra-articularly to rats with osteoarthritis, these hybrid CAP-exosomes (hybrid CAP-Exo/CRISPR/Cas9 sgMMP–13) penetrated deep into the cartilage matrix, delivered the plasmid to chondrocytes, and knocked down the expression of matrix metalloproteinase 13 (MMP–13) (Figure 8C). This targeted knockdown mitigated cartilage degradation and thereby demonstrated the potential of this delivery system to alleviate osteoarthritis [118]. Usman et al. presented a novel approach using human RBC-derived extracellular vesicles (RBCEVs) for the delivery of RNA drugs. It addressed the issues of low uptake efficiency and high cytotoxicity associated with current methods. RBCs, particularly group O-RBCs, are preferable for large-scale EV production owing to their availability, absence of DNA, and safety profiles. RBCEVs effectively delivered various RNA therapies including antisense oligonucleotides, CRISPR/Cas9 mRNA, and guide RNAs. As represented in Figure 8D, these demonstrated high efficiency in microRNA inhibition and CRISPR/Cas9 genome editing with no observed cytotoxicity. Unlike EVs from other sources, RBCEVs provide advantages in terms of scalability, safety, and stability. This makes these a potential platform for therapeutic RNA delivery that is potentially applicable to both common and rare targets [73]. Zhang et al. developed an efficient method for delivering large biomolecules into cells using “Gectosomes”. These are engineered ectosomes co-encapsulating VSV-G protein and bioactive macromolecules via split GFP complementation. This approach enabled active cargo loading, enhanced delivery specificity, and simplified gectosomal purification. Gectosomes reduce the non-specific encapsulation of cellular proteins. This allows for effective in vitro and in vivo gene editing and RNA interference. These leveraged VSV-G to efficiently cross the cell and endosomal membranes. This ensured the timely release of the cargo inside the cell. The method is advantageous for long-term gene editing and vaccine development. However, challenges, such as vesicle heterogeneity and immune responses to VSV-G or non-human proteins, remain. This necessitates further research [72].

Exosome-based nanoparticles provide a potential approach for CRISPR/Cas9 delivery, and a safe and efficient method for genome editing. As our understanding of nanocarrier technology and exosome biology continues to advance, these carriers hold considerable potential for transforming the CRISPR/Cas9 technology into clinical applications.

## 8. Conclusions and Future Perspectives

CRISPR/Cas9 has significantly advanced genetic engineering by providing a highly accurate and efficient method for targeted DNA modification. Initially, viral vectors such as adenoviral (AdV), adeno-associated viral (AAV), and lentiviral (LV) vectors were utilized for CRISPR/Cas9 delivery. Although effective, these viral vectors have limitations including off-target effects and immune responses. To address these challenges, researchers have focused increasingly on non-viral delivery systems for CRISPR/Cas9.

Non-viral vectors encompass a range of platforms, each with distinct characteristics and advantages. For example, lipid-based nanoparticles provide remarkable biocompatibility and efficient nucleic acid encapsulation. This makes these suitable carriers for CRISPR/Cas9. Similarly, polymer-based nanoparticles allow for diverse design options. This enables controlled release and target-specific delivery. Inorganic nanoparticles such as gold and iron oxide provide multifunctional capabilities and imaging functions. EV-based nanoparticles leverage natural in vivo delivery mechanisms.

Notwithstanding these advances, challenges remain in achieving precise targeting and minimizing off-target effects. The strategies to improve targeting include the surface modification of nanoparticles with cell-specific ligands, rational design of tissue-specific promoters for CRISPR/Cas9 expression, and engineering of gRNA for enhanced specificity. Ensuring the safety of CRISPR-based therapies is crucial. It necessitates the development of conditional expression systems and rigorous preclinical evaluations.

Although the translation of CRISPR/Cas9 technology into clinical applications presents ongoing challenges, its therapeutic potential remains substantial. Continuous research and development of non-viral vector-based delivery systems and nanocarrier platforms are critical for overcoming these obstacles and realizing the clinical advantages of CRISPR/Cas9 in gene therapy and precision medicine.

## Figures and Tables

**Table 1:** Orthologues of CRISPR/Cas9.

Name	Bacteria Species	PAM Site	Gene Size	Reference
SpCas9	*Streptococcus pyogenes*	5′-NGG-3′	4.2 kb	[33]
SaCas9	*Staphylococcus aureus*	5′-NNGRRT-3′	3.1 kb	[33]
St1Cas9	*Streptococcus thermophilus* LMD-9	5′-NNAGAAW-3′	3.3 kb	[33]
NmCas9	*Neisseria meningitides*	5′-NNNNGATT-3′	4.2 kb	[29]
SjCas9	*Campylobacter jejuni*	5′-NNNVRYM-3′	3.3 kb	[31]

**Table 2:** Clinical trials using CRISPR/Cas9.

Disease	Disease Condition	Target Gene	Intervention	Method	Reference
β-thalassemia	β-thalassemia (β^0^/β^0^, β^+^/β^0^, β^E^/β^0^)	*BCL11A*	Gene disruption	CRISPR/Cas9	[33]
SCD (Sickle cell disease)	SCD (β^s^/β^s^, β^s^/β^c^, β^s^/β^0^)	*HBB*	Gene disruption	CRISPR/Cas9	[33]
β-thalassemia and SCD	Transfusion-dependent β-thalassemia (β^0^/β^0^, β^+^/β^0^, β^E^/β^0^, β^+^/β^+^)	*BCL11A*	Gene disruption	CRISPR/Cas9	[33]

**Table 4:** Non-viral delivery system using nanoparticles for CRISPR/Cas9.

Type of Delivery System	Nanoparticle Formulation	CRISPR/Cas9 Cargo	Efficiency	Application	Reference
Lipid-based nanoparticle	polyethylene glycol phospholipid-modified cationic lipid nanoparticle (PLNP)	gRNA and Plasmid DNA encoding CRISPR/Cas9	67%>	In vitro, In vivo	[60]
	pegylated lipid and ADP–2k	iGeoCRISPR/Cas9	35–56% efficiency in the liver or lungs	In vitro, In vivo	[61]
	Cholesterol, C14-PEG 2000, DOPE and ionizable lipid cKK-E12	Pcsk9 (gRNA and CRISPR/Cas9 mRNA)	>80% editing of Pcsk9 in the liver	In vitro, In vivo	[62]
	Zwitterionic amino lipids (ZALs),	CRISPR/Cas9/gRNA mRNA	95 % decrease in protein expression (HeLa-Luc-CRISPR/Cas9)	In vitro, In vivo	[63]
	Biodegradable ionizable lipid (LP01), cholesterol, DSPC and PEG2k-DMG	CRISPR/Cas9/gRNA mRNA	>97% reduction in the liver	In vitro, In vivo	[55]
Polymer-based nanoparticle	PEG_5K_-b-PLGA_11K_	Plasmid DNA (pCRISPR/Cas9)	74.6% (EGFP-positive K562 cells for CLANpCRISPR/Cas9-EGFP)	In vitro	[64]
	Cationic polymer polyethylenimine (PEI) coated self-assembled DNA nanoclews	CRISPR/Cas9/gRNA RNP	80% (EGFP in U2OS)	In vitro	[65]
	Graphene oxide (GO)-polyethylene glycol (PEG)-polyethylenimine (PEI) nanocarrier	CRISPR/Cas9/gRNA RNP	∼39% (gene editing in human AGS cells with an efficiency)	In vitro	[58]
	Carboxymethyl chitosan (biotinylated carboxymethyl chitosan with biotin ligands and aptamer-incorporated carboxymethyl chitosan with AS1411 ligands)	pDNA	>90% (CDK_11_ protein)	In vitro	[66]
	PEGylated nanoparticles (named P-HNPs) based on the cationic α-helical polypeptide poly(γ–4–((2-(piperidin–1–yl)ethyl)aminomethyl)benzyl-_L_-glutamate)	CRISPR/Cas9/gRNA pDNA	60% (CRISPR/Cas9 transfection efficiency), 67.4% (gRNA uptake efficiency), >71% (suppressing the tumor growth)	In vitro, In vivo	[59]
Inorganic nanoparticles	CRISPR-Gold	RNP	40–50% (Reduced mRNA levels and the protein levels of mGluR5)	In vitro, In vivo	[67]
	CRISPR-Gold	RNP	61.5% (encapsulation efficiency), 11.3% (BFP-HEK cells to express GFP via HDR)	In vivo	[68]
	Complexing magnetic nanoparticles (MNPs) with recombinant baculoviral vectors (BVs)	Recombinant baculoviral vector (BV)	~50% indel rate in mouse liver cells	In vitro, In vivo	[69]
	monosized lipid-coated mesoporous silica nanoparticle (LC-MSN)	RNP	70% (release within cancer cells), 10% (gene editing)	In vitro, In vivo	[70]
	Lipid-coated mesoporous silica nanoparticles (LCMSNs)	RNP	20% (Edition against both targets)	In vitro	[71]
Extracellular Vesicles-Based nanoparticles	Gectosomes (Virus G protein; VSV-G, split GFP)	CRISPR/Cas9 protein and RNP	40% (Reduction in the number of cells positive for Parkin recruitment)	In vitro, In vivo	[72]
	Red blood cells (RBCs)-derived EV	CRISPR/Cas9 mRNA	~32% (loss of eGFP in ~32% NOMO1-eGFP cells)	In vitro, In vivo	[73]
	Non-autologous exosomes	CRISPR/Cas9 plasmid DNA	~58% suppression (moderate knockdown)	In vitro, In vivo	[74]
	Tumor-derived exosomes (SKOV3-Exo)	CRISPR/Cas9/gRNA plasmid DNA	27% (Indel at electroporated into SKOV3-Exo), 57% (Inhibited the cellular proliferation at co-treatment with cisplatin and iPARP–1/SKOV3-Exo)	In vitro, In vivo	[75]
